# Styptic Colloid

**Published:** 1871-05

**Authors:** 


Styptic Colloid, for promoting the healing of wounds by the first intention, or for treating open and fœtid wounds, and for arresting hemorrhage, is well known to the profession. We have found the colloid a very good application for herpes zoster. Mr. Browne (*Liverpool Hospital Reports*, vol. iv) recommends us to paint a patch of eczema, complicated with varicose veins, freely with Richardson's styptic colloid, allowed to evaporate to half its bulk before being used; the film left by the colloid dries and contracts; it is then to be covered with a sheet of thin vulcanized india-rubber, and bandaged. Thus pressure is applied, and the atmosphere excluded.—*Journal of Cutaneous Medicine.*

